# CCAAT/Enhancer Binding Protein β Regulates Expression of Indian Hedgehog during Chondrocytes Differentiation

**DOI:** 10.1371/journal.pone.0104547

**Published:** 2014-08-08

**Authors:** Takahiro Ushijima, Ken Okazaki, Hidetoshi Tsushima, Kohei Ishihara, Toshio Doi, Yukihide Iwamoto

**Affiliations:** Department of Orthopaedic Surgery, Graduate School of Medical Sciences, Kyushu University, Fukuoka, Japan; Università degli Studi di Milano, Italy

## Abstract

**Background:**

CCAAT/enhancer binding protein β (C/EBPβ) is a transcription factor that promotes hypertrophic differentiation of chondrocytes. Indian hedgehog (Ihh) also stimulates the hypertrophic transition of chondrocytes. Furthermore, runt-related transcription factor-2 (RUNX2) was reported to regulate chondrocyte maturation during skeletal development and to directly regulate transcriptional activity of *Ihh*. In this study, we investigated whether the interaction of C/EBPβ and RUNX2 regulates the expression of *Ihh* during chondrocyte differentiation.

**Methodology/Results:**

Immunohistochemistry of embryonic growth plate revealed that both C/EBPβ and Ihh were strongly expressed in pre-hypertrophic and hypertrophic chondrocytes. Overexpression of C/EBPβ by adenovirus vector in ATDC5 cells caused marked stimulation of *Ihh* and *Runx2*. Conversely, knockdown of C/EBPβ by lentivirus expressing shRNA significantly repressed *Ihh* and *Runx2* in ATDC5 cells. A reporter assay revealed that C/EBPβ stimulated transcriptional activity of *Ihh*. Deletion and mutation analysis showed that the C/EBPβ responsive element was located between −214 and −210 bp in the *Ihh* promoter. An electrophoretic mobility shift assay (EMSA) and a chromatin immunoprecipitation (ChIP) assay also revealed the direct binding of C/EBPβ to this region. Moreover, reporter assays demonstrated that RUNX2 failed to stimulate the transcriptional activity of the *Ihh* promoter harboring a mutation at the C/EBPβ binding site. EMSA and ChIP assays showed that RUNX2 interacted to this element with C/EBPβ. Immunoprecipitation revealed that RUNX2 and C/EBPβ formed heterodimer complex with each other in the nuclei of chondrocytes. These data suggested that the C/EBPβ binding element is also important for RUNX2 to regulate the expression of *Ihh*. *Ex vivo* organ culture of mouse limbs transfected with C/EBPβ showed that the expression of Ihh and RUNX2 was increased upon ectopic C/EBPβ expression.

**Conclusions:**

C/EBPβ and RUNX2 cooperatively stimulate expression of *Ihh* through direct interactions with a C/EBPβ binding element, which further promotes hypertrophic differentiation of chondrocytes during the chondrocyte differentiation process.

## Introduction

Chondrocyte differentiation and hypertrophic transition are crucial processes not only for skeletal formation, but also during osteoarthritis (OA) development [1]–[3]. Chondrogenesis is initiated when mesenchymal cells condense and differentiate into proliferative chondrocytes. Thereafter, the chondrocytes change their morphology to become pre-hypertrophic and hypertrophic chondrocytes. Finally, osteoblast and osteoclast precursors migrate into the cartilage, which is accompanied by vascular invasion and apoptosis of mature hypertrophic chondrocytes to complete the formation of bone. This process is known as endochondral ossification.

Differentiation from proliferative to hypertrophic chondrocytes is a dynamic change in terms of morphology and biochemistry [3]. The differentiation process is tightly regulated by various factors such as locally secreted factors and transcription factors. Among these factors, Indian hedgehog (Ihh), which is a member of the hedgehog family, was reported to be involved in this regulation. Ihh, which is expressed by pre-hypertrophic chondrocytes, diffuses to the cells in the articular perichondrium where it stimulates expression of parathyroid hormone related protein (PTHrP), which negatively regulates hypertrophic differentiation [4]. This is the so-called Ihh/PTHrP negative feedback loop, which strictly regulates the pace of differentiation from proliferative to hypertrophic chondrocytes. Furthermore, Ihh itself was shown to promote hypertrophic differentiation of chondrocytes by activating Wingless-type MMTV integration site (Wnt)/β-catenin and bone morphogenetic protein (BMP) signaling [5], [6].

C/EBP is a family of basic leucine zipper transcription factors with 6 members as follows: C/EBPα, β, δ, ε, γ, and ζ. Among them, C/EBPβ (encoded by *CEBPB*) was first identified as a nuclear protein that bound to an IL-1β response element in the IL-6 promoter region [7] and it was subsequently reported to regulate various genes involved in cell differentiation, proliferation, survival, immune function, tumor invasiveness and progression [8]–[11]. C/EBPβ has three major isoforms: 38 kD (liver-enriched activator protein Star [LAP*]), 36 kD (LAP) and 20 kD (liver-enriched inhibitory protein [LIP]) [10], [12]. We previously reported that C/EBPβ, in response to IL-1β, down-regulated cartilage-derived retinoic acid-sensitive protein (Cd-rap) [13]. C/EBPβ stimulates the expression of matrix metalloproteinases (MMP) 3 and MMP13 in arthritic cartilage such as osteoarthritis and rheumatoid arthritis [14], [15]. C/EBPβ was also reported to promote the differentiation from proliferative to hypertrophic chondrocytes by enhancing the expression of p57, type X collagen (COL10A1) and MMP13 [16]–[18]. Recently, we reported that C/EBPβ repressed the expression of type II collagen (COL2A1) and sex-determining region Y-type high mobility group box 9 (SOX9) during chondrocyte differentiation [19]. Thus, C/EBPβ has multiple functions and is a crucial transcription factor that regulates the differentiation from proliferative to hypertrophic chondrocytes.

C/EBPβ also interacts with other transcription factors. During skeletal development, C/EBPβ was reported to stimulate MMP13 and osteocalcin expression cooperatively with runt-related transcription factor-2 (RUNX2) [17], [20], [21]. This is a transcription factor that regulates chondrocyte maturation and osteoblast differentiation [22]. Furthermore, it was reported that RUNX2 directly regulates the expression of *Ihh* by interacting with its promoter region during chondrocyte differentiation [23].

Although both C/EBPβ and Ihh were reported to stimulate hypertrophic differentiation of chondrocytes, the interaction between them remains unknown. Here, we demonstrate that C/EBPβ and RUNX2 cooperatively stimulate expression of *Ihh* through direct interactions with its promoter region during chondrocyte differentiation.

## Materials and Methods

### Ethics statement

Experiments using mice tissue samples were performed in compliance with the guideline established by the Animal Care and Use Committee of the Kyushu University. The protocol was approved by the Committee on the Ethics of Animal Experiments of the Kyushu University (Permit Number: A25-186).

### Immunohistochemistry

Tissue samples of growth plate were obtained from mouse embryos (E16.5). For immunoperoxidase method, Vectastain Elite ABC kit (Vector Laboratories; Burlingame, CA) was used. Deparaffinized sections (3 µm thickness) were subjected to antigen retrieval by microwaving in 10 mM citrate buffer (sodium citrate, pH 6.0) for 20 minutes. Endogenous peroxidase activity was blocked by incubation in 3% H_2_O_2_ in methanol for 30 minutes. The specimens were placed in blocking reagent for 30 minutes and incubated overnight at 4°C with the following primary antibodies: C/EBPβ (C-19; Santa Cruz Biotechnology, Santa Cruz, CA) diluted 1∶500, RUNX2 (AP7735a; Abgent, San Diego, CA) diluted 1∶200, Ihh (C-15; Santa Cruz Biotechnology), or normal rabbit IgG (sc-2027; Santa Cruz Biotechnology) diluted 1∶1000. The samples were further incubated with secondary antibodies for 30 minutes and then a colorimetric reaction was carried out with 3,3′-diaminobenzidine and 0.02% H_2_O_2_, followed by counterstaining with hematoxylin. For immunofluorescent staining, Alexa Fluor 568 (Invitrogen, Carlsbad, CA) were used as a secondary antibody and mounted with VECTASHIELD Mounting Medium with DAPI (Vector Laboratories).

### Cell culture

ATDC5 cells (RIKEN cell bank, Tsukuba, Japan), a mouse chondrogenic cell line, were maintained in Dulbecco's modified Eagle's medium (DMEM)/Ham's F-12 medium supplemented with 5% fetal bovine serum (FBS). To induce differentiation, subconfluent cultures were changed to medium containing 1% ITS (insulin–transferrin–selenium) Universal Culture Supplement Premix reagent (BD Biosciences) [24]. HeLa cells were cultured with DMEM containing 10% FBS.

### Virus vectors

Adenovirus vectors expressing C/EBPβ-LAP or LacZ control were kindly provided by Dr. Hiroshi Sakaue (Kobe University, Kobe, Japan) [25]. LAP is one of the isoforms of C/EBPβ, which carries a trans-activator domain [12]. ATDC5 cells were transfected with these vectors and differentiated for 2 weeks with ITS. Stable ATDC5 cell lines were generated with lentivirus vectors expressing short hairpin RNA (shRNA) for *Cebpb* (TRCN0000231407) (Sigma Aldrich, St. Louis, MO) or control. ATDC5 cells selected with puromycin (2 µg/ml) were differentiated for 2 weeks with ITS.

### RNA extraction, quantitative real-time RT-PCR and semiquantitative RT-PCR

Total RNA was isolated from cultured cells using the RNeasy mini kit (Qiagen, Hilden, Germany) and was reverse-transcribed using the Prime script RT reagent kit (Takara Bio, Shiga, Japan) to make single-stranded cDNA. Quantitative real-time RT-PCR was performed with the Light Cycler 2.0 System (F. Hoffmann-La Roche AG, Basel, Switzerland) using SYBR Premix Ex Taq II (Takara Bio). The primers were as follows: for *Cebpb*, 5′- ACGACTTCCTCTCCGACCTCT -3′ (forward) and 5′- CGAGGCTCACGTAACCGTAGT -3′ (reverse); *Runx2*, 5′- AACCACAGAACCACAAGT -3′ (forward) and 5′- AAATGACTCGGTTGGTCT -3′ (reverse); for *Ihh*, 5′- GACTCATTGCCTCCCAGAACTG -3′ (forward) and 5′- CCAGGTAGTAGGGTCACATTGC -3′ (reverse); for *Pthrp*, 5′- ACTCCTTCCAGGGATTTTTTGTT -3′ (forward) and 5′- GAAGTCCAATGCCAGTGTCCA -3′ (reverse); and for *18S*, 5′- GTAACCCGTTGAACCCCATT -3′ (forward) and 5′- CCATCCAATCGGTAGTAGCG -3′ (reverse). Data were corrected for expression of the housekeeping gene *18S*.

### Western blot

Nuclear extracts were isolated using Nuclear and Cytoplasmic Extraction Reagents (Pierce, Rockford, IL). Cell lysates were electrophoresed in 4–12% gradient polyacrylamide gels (Invitrogen) and transferred to nitrocellulose membranes (Amersham, Arlington Heights, IL). After blocking in Tris-buffered saline-Tween containing 3% non-fat milk, the membranes were incubated with primary antibodies against C/EBPβ diluted 1∶300, RUNX2 (M-70; Santa Cruz Biotechnology) diluted 1∶200 in blocking reagent at room temperature for 1 hour. We also used anti-LAMIN A/C (H-110; Santa Cruz Biotechnology) antibodies as internal loading controls. Horseradish peroxidase-conjugated secondary antibody (Santa Cruz Biotechnology) diluted in blocking reagent was added and incubated at room temperature for 1 hour. The immunoreactivity of the blots was detected using ECL Prime (Amersham).

### Plasmid preparation and reporter assay

Mouse *Ihh* sequences spanning from −1224 to +43 bp were subcloned into the pGL-4.10 (luc2) vector (Promega, Madison, WI). Deletion sequences were also generated by PCR technique. Site-directed mutagenesis was performed using KOD Plus Mutagenesis Kit (Toyobo, Osaka, Japan). These plasmids were co-transfected into HeLa cells using Lipofectamine 2000 reagent (Invitrogen) with expression vectors as follows: pCMV-LAP (an expression vector of rat C/EBPβ), an A-C/EBP vector tagged with Flag (a dominant-negative C/EBP expression vector kindly provided by Dr. Charles R. Vinson) and RUNX2 expression vector (kindly provided by Dr. Toshihisa Komori [26]). Reporter activity was measured 48 hours after transfection using the Dual-Luciferase Reporter Assay System (Promega).

### Electrophoretic mobility shift assay (EMSA)

Nuclear protein was extracted from ATDC5 cells that had been transfected with C/EBPβ. Complementary oligonucleotides were end-labeled with the Biotin 3′ End DNA Labeling Kit (Thermo Scientific), then annealed to obtain double-stranded oligonucleotides. EMSA was performed using the LightShift Chemiluminescent EMSA Kit (Thermo Scientific). Twenty fmol of biotin-labeled probes were incubated with nuclear protein in 1× binding buffer (including 2.5% glycerol, 5 mM MgCl_2_, 50 ng/µl poly(dI-dC)) at room temperature for 20 minutes. For competition experiments, the cold probes were added at a 200-fold molar excess. For antibody interference experiments, the nuclear extract was pre-incubated with 1 µl of C/EBPβ, RUNX2 (M-70) or IgG antibody for 1 hour at 4°C. Binding samples were subjected to electrophoresis in a 6% DNA Retardation gel (Invitrogen) and run in 0.5× TBE buffer at 100 V for 1 hour, then transferred to a positively charged membrane (Invitrogen) and cross-linked. Detection was performed using streptavidin-horseradish peroxidase conjugate and chemiluminescent substrate. The oligonucleotides were as follows: wild-type, 5′- GGCCTATTTATTGGCGGCCGGCG -3′ (sense) and 5′- CGCCGGCCGCCAATAAATAGGCC -3′ (antisense); and mutant, 5′- GGCCTATTTCGCGGCGGCCGGCG -3′ (sense) and 5′- CGCCGGCCGCCGCGAAATAGGCC -3′ (antisense).

### Chromatin immunoprecipitation (ChIP) assay

ChIP assay was performed with a ChIP Assay kit (Millipore). ATDC5 cells were differentiated for 3 weeks to induce hypertrophic differentiation. The ATDC5 cells were fixed with 4% formaldehyde and sonicated. For immunoprecipitation, C/EBPβ, RUNX2 or normal rabbit IgG was used. Primers used in PCR were as follows: amplified between −259 and −160 bp for *Ihh* promoter including the C/EBPβ binding motifs, and between −1274 and −1102 bp as a negative control. The PCR products were amplified for 35 cycles.

### Immunoprecipitation (IP)

Nuclear protein was extracted from ATDC5 cells that had been transfected with the C/EBPβ expression vector. IP was performed with an Immunoprecipitation kit (Invitrogen) according to the manufacturer's instructions. For immunoprecipitation, nuclear extract was incubated with magnetic beads conjugated with C/EBPβ, RUNX2 or normal rabbit IgG antibody for 10 minutes. Analysis was performed by immunoblotting.

### *Ex vivo* organ culture

Tibias were isolated from hind limbs of E14.5 mouse embryos and cultured in organ culture medium. One day after dissection, each tibia obtained from identical mouse embryos were transfected with adenovirus vectors expressing C/EBPβ-LAP or LacZ control and cultured at 37°C in a humidified 5% CO_2_ incubator for 4 days. Safranin O and immunofluorescent staining was performed. Histological analysis was repeated at least twice for each sample from six pairs of limbs, respectively.

### Statistical analysis

Data are reported as mean ± S.D. of three independent experiments, each performed in duplicate. Data analysis was performed using statistical software JMP 9 (SAS Institute, Inc. Cary, NC). The Mann-Whitney U-test was used for two-group comparisons. *p*<0.05 was considered statistically significant.

## Results

### Expression patterns of C/EBPβ, RUNX2 and Ihh *in vivo*

**Figure 1:**
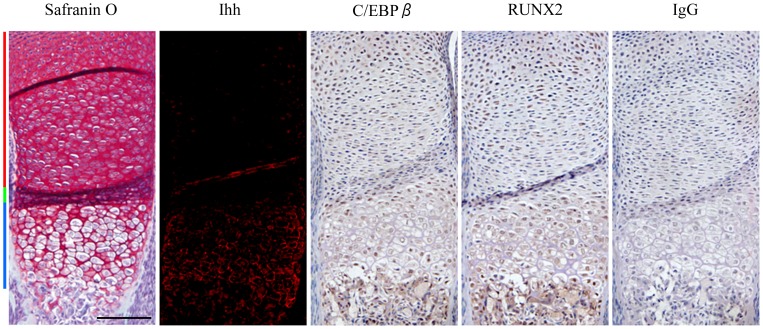
Expression patterns of C/EBPβ, RUNX2 and Ihh during chondrocyte differentiation. Upper limbs obtained from mouse embryos (E16.5) were subject to immunohistochemistry with Ihh, C/EBPβ and RUNX2 antibodies. Tissue stained with IgG is shown as a negative control. Hematoxylin was used as a counterstain. Red, green and blue bars indicate the proliferative, pre-hypertrophic and hypertrophic zones, respectively. Scale bar, 500 µm. Data are representative of two independent experiments performed in duplicate.

To confirm the endogenous expression of C/EBPβ, RUNX2 and Ihh, immunohistochemistry was performed using upper limbs obtained from E16.5 mice embryos (Figure 1). Both C/EBPβ and RUNX2 were weakly expressed by proliferative chondrocytes, but strongly expressed by pre-hypertrophic and hypertrophic chondrocytes. Similarly, Ihh expression was detected in pre-hypertrophic and hypertrophic chondrocytes. The similar distribution of C/EBPβ and Ihh in the growth plate suggested that C/EBPβ could be involved in the regulation of Ihh during differentiation from proliferative to hypertrophic chondrocytes.

### C/EBPβ stimulates expression of *Ihh* during chondrocyte differentiation

**Figure 2:**
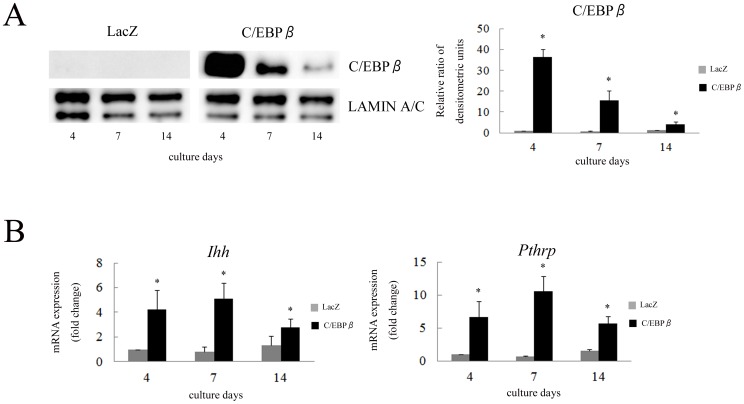
C/EBPβ stimulated the expression of *Ihh* and *Runx2* in ATDC5 cells. (A) Western blot of nuclear extracts obtained from ATDC5 cells, which were transfected with LacZ or C/EBPβ-LAP, was performed to investigate the expression of C/EBPβ. Densitometric scanning of C/EBPβ expression was performed. Each density of C/EBPβ was normalized with that of LAMIN A/C and the ratio by corrected densities of C/EBPβ to control on the 4th day was calculated. Data are representative of two independent experiments performed in duplicate. ^*^ *p*<0.05 vs. LacZ. (B) ATDC5 cells were differentiated for 2 weeks after transfection with adenovirus vectors expressing C/EBPβ-LAP and LacZ control. Expression of *Ihh* and *Pthrp* mRNA was determined by real-time RT-PCR. Each value was normalized to *18S* in the same sample. The value of each mRNA expression relative to that of LacZ on the 4th day was indicated. Means ± S.D. of duplicates from three independent experiments are shown. ^*^ *p*<0.05 vs. LacZ.

To investigate the effect of C/EBPβ on *Ihh* expression, ATDC5 cells were transfected with adenovirus vectors expressing C/EBPβ-LAP or LacZ control and the cells were differentiated for 2 weeks. Increase of the mRNA (not shown) and nuclear protein of C/EBPβ-LAP by infection of adenovirus vector demonstrated that transfection of C/EBPβ was effectively performed (Figure 2A). We previously reported that in the same model, exogenous C/EBPβ significantly increased the expression of *Runx2* on the 4th and 7th days [19]. The expression of *Ihh* was significantly increased at all the differentiation stages (Figure 2B). The expression of *Pthrp*, which is regulated by Ihh, was also stimulated by overexpression of C/EBPβ.

**Figure 3:**
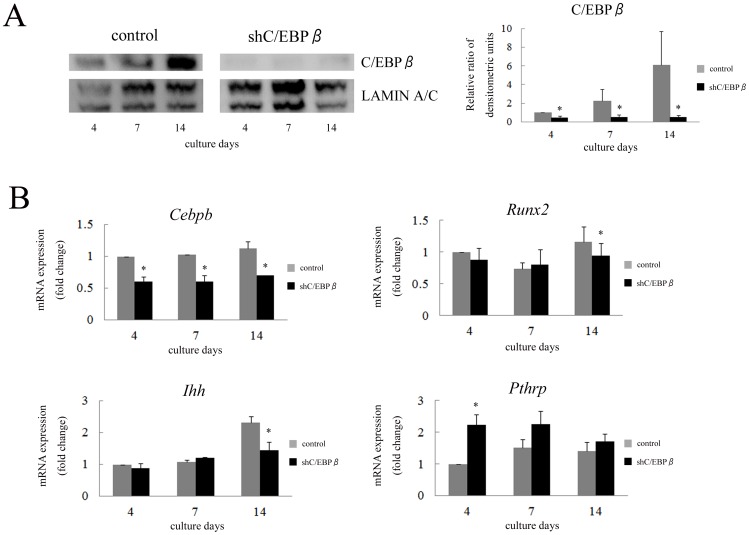
Knockdown of C/EBPβ repressed the expression of *Ihh* and *Runx2* in ATDC5 cells. (A) Western blot of nuclear extracts obtained from stable ATDC5 cells was performed to investigate the expression of C/EBPβ. Densitometric scanning of C/EBPβ expression was performed. Each density of C/EBPβ was normalized with that of LAMIN A/C and the ratio by corrected densities of C/EBPβ to control on the 4th day was calculated. Data are representative of two independent experiments performed in duplicate. ^*^ *p*<0.05 vs. control. (B) ATDC5 cells stably expressing shRNA for *Cebpb* were differentiated for 2 weeks. Expression of *Cebpb*, *Runx2*, *Ihh* and *Pthrp* mRNA was determined by real-time RT-PCR. Each value was normalized to *18S* in the same sample. The value of each mRNA expression relative to that of control on the 4th day was indicated. Means ± S.D. of duplicates from three independent experiments are shown. ^*^ *p*<0.05 vs. control.

Next, we investigated the effect of C/EBPβ knockdown on the expression of *Ihh*. ATDC5 cells were transfected with lentivirus expressing shRNA targeting *Cebpb* and stably infected cells were differentiated with ITS for 2 weeks. Knockdown of *Cebpb* was confirmed with nuclear extracts and mRNA in cells transfected with shRNA compared to the controls at all differentiation stages (Figure 3A, B). *Ihh* and *Runx2* expression was significantly repressed by shRNA for *Cebpb* on the 14th day (Figure 3B). However, the expression of *Pthrp* was markedly increased by shRNA on the 4th day (Figure 3B). These results suggest that C/EBPβ is involved in the regulation of *Ihh* expression at the endogenous level during chondrocyte differentiation.

### C/EBPβ up-regulates transcriptional activity of *Ihh*

**Figure 4:**
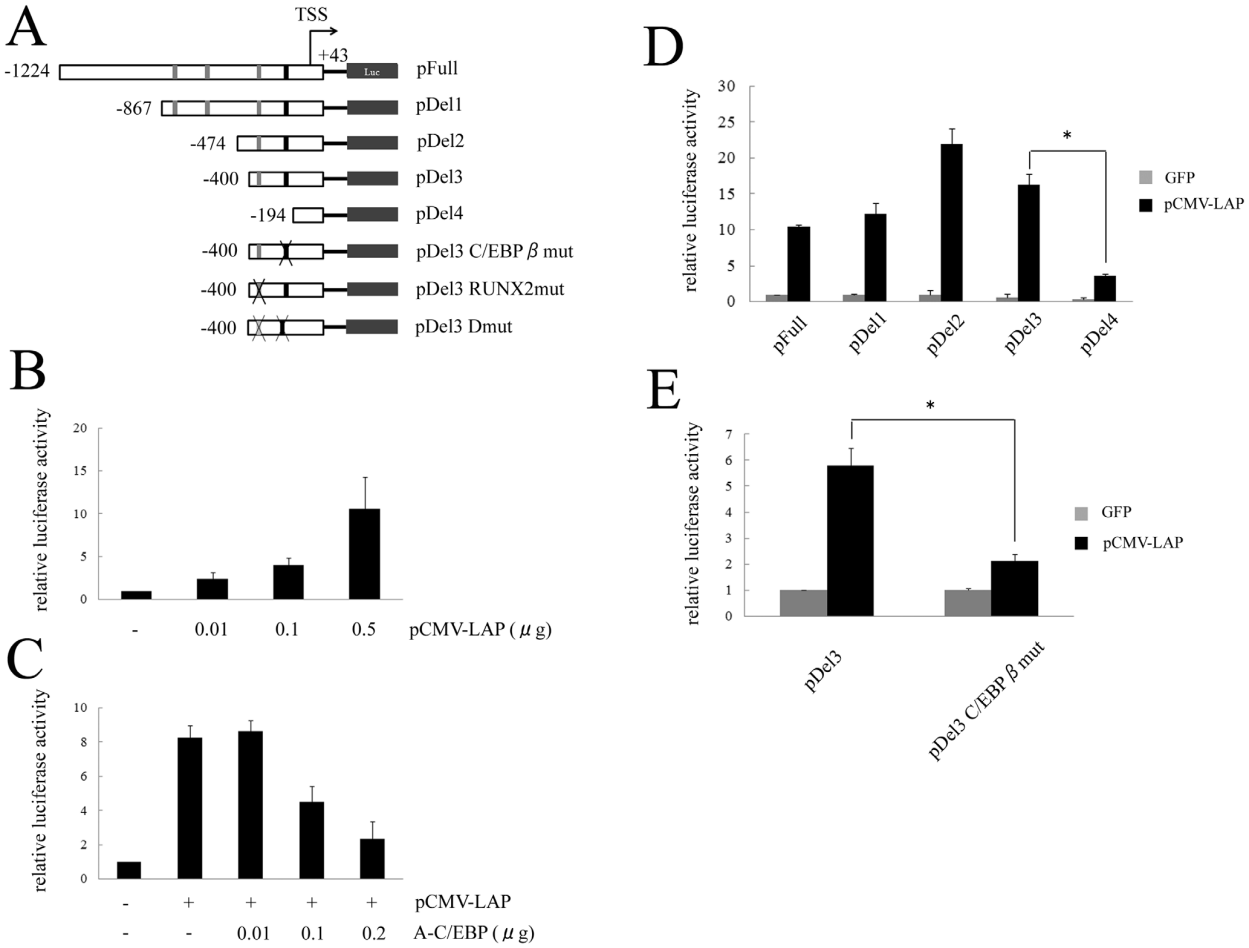
C/EBPβ up-regulated transcriptional activity of *Ihh*. (A) The *Ihh* reporter construct containing −1224 to +43 bp of the *Ihh* promoter, and various deletion constructs were generated. Gray and black boxes indicate RUNX2 binding elements reported by a previous study and C/EBPβ binding motif, respectively. Mutation construct of each element was also generated. (B) The *Ihh* reporter construct (pFull) was co-transfected with pCMV-LAP and GFP into HeLa cells. Means ± S.D. of duplicates from three independent experiments are shown. (C) The *Ihh* reporter construct (pFull) was co-transfected with 0.1 µg of pCMV-LAP and various amounts of A-C/EBP into HeLa cells. Means ± S.D. of duplicates from three independent experiments are shown. (D) Deletion constructs were co-transfected with 0.1 µg of pCMV-LAP or GFP into HeLa cells. Means ± S.D. of duplicates from three independent experiments are shown. ^*^ *p*<0.05. (E) A mutation construct of C/EBPβ binding motif in pDel3 was co-transfected with 0.1 µg of pCMV-LAP or GFP into HeLa cells. Means ± S.D. of duplicates from three independent experiments are shown. ^*^ *p*<0.05.

To confirm the transcriptional regulation of *Ihh* by C/EBPβ, a luciferase reporter construct containing −1224 to +43 bp of the *Ihh* promoter was generated (Figure 4A) and it was co-transfected with various expression vectors into HeLa cells. C/EBPβ up-regulated *Ihh* promoter activity in a dose-dependent manner (Figure 4B). In contrast, A-C/EBP, which inhibits binding of C/EBP family members to specific binding sites by forming a heterodimeric complex [27], reversed the up-regulation of *Ihh* promoter activity caused by C/EBPβ in a dose-dependent manner (Figure 4C). These results suggest that C/EBPβ regulates the expression of *Ihh* at the transcriptional level.

### C/EBPβ stimulates expression of *Ihh* by directly binding to its promoter region

To identify the C/EBPβ response element in the *Ihh* gene, a series of 5′ promoter deletion constructs were generated (Figure 4A). C/EBPβ stimulated luciferase activity of the *Ihh* reporter construct when the promoter sequence was deleted to −400 bp (Figure 4D). However, C/EBPβ could not stimulate the luciferase activity of pDel4, demonstrating that a functional element for C/EBPβ was located between −400 and −194 bp in the *Ihh* promoter. Analysis of the sequence indicated the presence of one C/EBPβ binding motif in the promoter element. To further demonstrate transcriptional regulation by C/EBPβ at this binding motif, site-directed mutagenesis was performed. A point mutation in the C/EBPβ binding motif was introduced into the pDel3 construct (Figure 4A). Promoter activity of pDel3-C/EBPβmut by C/EBPβ was markedly decreased compared with that of pDel3 (Figure 4E). These results suggest that C/EBPβ stimulates the expression of *Ihh* by interacting with its promoter region.

**Figure 5:**
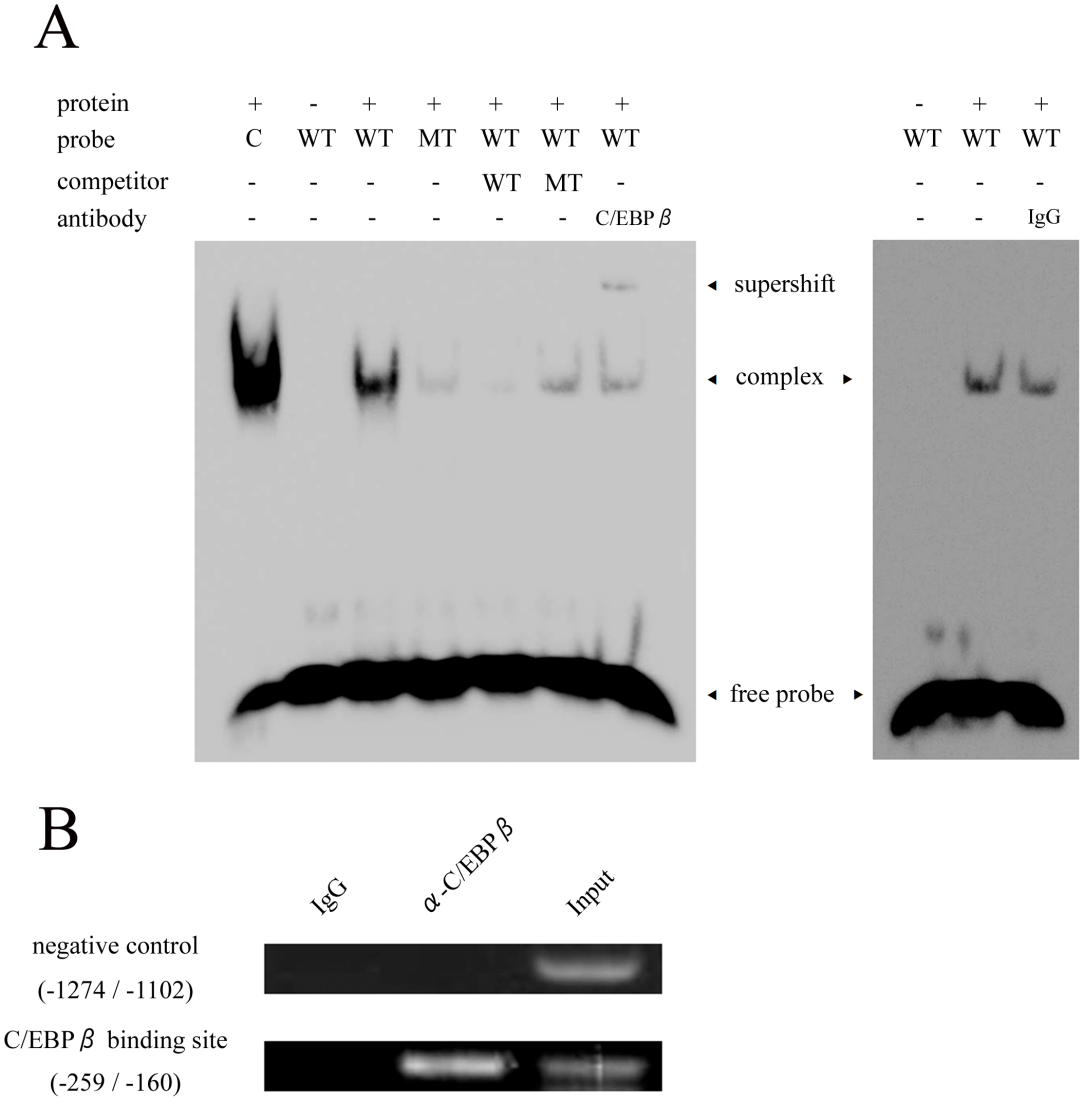
C/EBPβ directly bound to *Ihh* promoter. (A) EMSA for specific binding of C/EBPβ to the *Ihh* promoter. Consensus oligonucleotide (C), wild-type (WT) and mutant (MT) probes were incubated with nuclear extract from C/EBPβ-transfected ATDC5 cells. Competition and supershift experiments were also performed. Data are representative of two independent experiments performed in duplicate. (B) A ChIP assay for C/EBPβ using ATDC5 cells cultured for 3 weeks. Semi-quantitative RT-PCR was performed using primers as follows: promoter region of *Ihh* (from −259 to −160) and negative control (from −1274 and −1102 bp). Data are representative of two independent experiments performed in duplicate.

To confirm the direct binding of C/EBPβ to the *Ihh* gene, EMSA was performed (Figure 5A). C/EBPβ bound strongly to the wild-type (WT) probe, but binding to the mutant (MT) probe was weak. Non-labeled WT probe inhibited the binding of C/EBPβ to labeled WT probe, but non-labeled MT probe could not block it. Supershift was observed by addition of a C/EBPβ antibody. Furthermore, a ChIP assay was performed using ATDC5 cells cultured for 3 weeks (Figure 5B). Endogenous C/EBPβ bound to the *Ihh* promoter region from −259 bp to −160 bp as detected by PCR. These analyses revealed a direct and specific binding of C/EBPβ to the *Ihh* promoter. Together, these results indicated that C/EBPβ directly stimulates transcriptional activity of *Ihh* by interacting with its promoter region.

### RUNX2 stimulates transcriptional activity of *Ihh* through its C/EBPβ binding element

**Figure 6:**
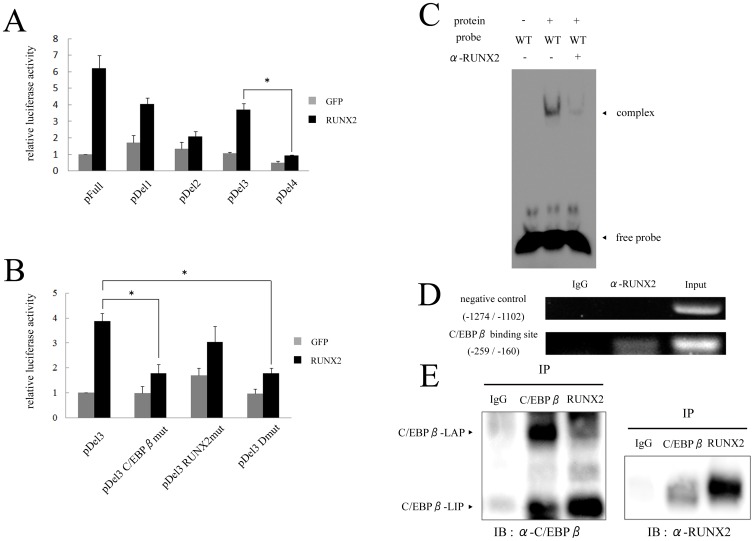
C/EBPβ binding element is crucial for RUNX2 to regulate transcriptional activity of *Ihh*. (A) Deletion constructs were co-transfected with 0.2 µg of RUNX2 or GFP into HeLa cells. Means ± S.D. of duplicates from three independent experiments are shown. ^*^ *p*<0.05. (B) Mutation constructs of C/EBPβ and RUNX2 binding elements in pDel3 were co-transfected with 0.2 µg of RUNX2 or GFP into HeLa cells. Means ± S.D. of duplicates from three independent experiments are shown. ^*^ *p*<0.05. (C) EMSA for specific binding of RUNX2 to the C/EBPβ binding site of *Ihh* promoter. Wild-type (WT) probe, which harbors C/EBPβ binding site, was incubated with nuclear extract from C/EBPβ-transfected ATDC5 cells. Supershift experiment using RUNX2 antibody was also performed. Data are representative of two independent experiments performed in duplicate. (D) A ChIP assay for RUNX2 using ATDC5 cells cultured for 3 weeks. Semi-quantitative RT-PCR was performed using same primers as indicated in Figure 5B. Data are representative of two independent experiments performed in duplicate. (E) Immunoprecipitation (IP) and Immunoblotting were performed. Nuclear extract was obtained from C/EBPβ-transfected ATDC5 cells. Immunoprecipitated proteins with C/EBPβ, RUNX2 or IgG antibody were subjected to SDS-PAGE and immunoblotting using C/EBPβ or RUNX2 antibody.

It has been reported that C/EBPβ regulates transcriptional activity of various genes by interacting with RUNX2 [17], [20], [21] and that RUNX2 directly regulates *Ihh* through its promoter region [23]. Therefore, we investigated the cooperative binding of C/EBPβ and RUNX2 in the regulation of *Ihh* expression. Similar to the results with C/EBPβ, RUNX2 stimulated the promoter activity of the *Ihh* deletion constructs until the promoter sequence was deleted to −400 bp (Figure 6A). A previous study reported that there were three RUNX2 binding sites in the *Ihh* promoter [23]. The pDel3 construct contains one functional binding site for RUNX2, which is located nearest to the transcription start site (Figure 4A). Interestingly, RUNX2 could not enhance the promoter activity of pDel3-C/EBPβmut even with a functional RUNX2 binding site (Figure 6B). In contrast, a point mutation introduced into the RUNX2 binding element in pDel3 (pDel3-RUNX2mut) had a weak effect on the promoter activity by exogenous RUNX2 (Figure 6B). As expected, RUNX2 could not stimulate the promoter activity of pDel3-Dmut, which had mutations in both the C/EBPβ and RUNX2 binding elements (Figure 6B). An EMSA revealed that the band intensity of the DNA probe for the sequence of the C/EBPβ binding site and protein complex was decreased when adding RUNX2 antibody (Figure 6C). In addition, a ChIP assay revealed binding of endogenous RUNX2 to the *Ihh* promoter located between −259 bp and −160 bp (Figure 6D). To confirm the interaction between C/EBPβ and RUNX2, IP was performed (Figure 6E). Immunoblotting with C/EBPβ antibody showed positive bands for C/EBPβ-LAP and –LIP on the sample immunoprecipitated with RUNX2 antibody. Immunoblotting with RUNX2 was also positive on the sample immunoprecipitated with C/EBPβ antibody. This result demonstrated that RUNX2 forms heterodimer complex with both of C/EBPβ-LAP and -LIP in the nuclei of chondrocytes. Together, these results indicated that the C/EBPβ binding site is also important for RUNX2 to regulate transcriptional activation of *Ihh*.

### Ectopic expression of C/EBPβ stimulates the expression of Ihh in *ex vivo* organ culture

**Figure 7:**
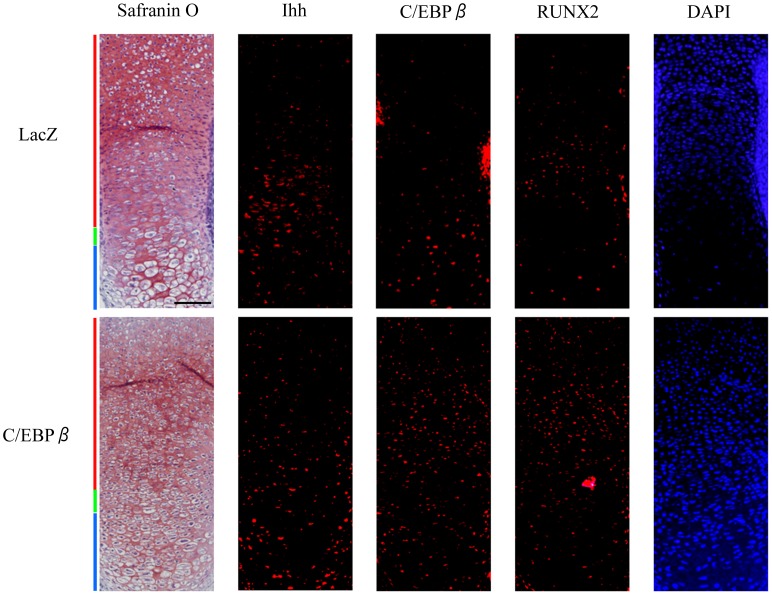
C/EBPβ stimulated the expression of Ihh in *ex vivo* organ cultures. *Ex vivo* organ culture of tibias dissected from E14.5 mouse embryos. Tibias were cultured for 4 days after transfection with adenovirus vectors expressing LacZ control (top row) and C/EBPβ-LAP (bottom row). Safranin O staining and immunofluorescent staining were performed to localize C/EBPβ, RUNX2 and Ihh. DAPI was used as a counterstain. Red, green and blue bars indicate the proliferative, pre-hypertrophic and hypertrophic zones, respectively. Scale bar, 500 µm. Histological analysis was repeated at least twice for each sample from six pairs of limbs, respectively.

Finally, we performed an *ex vivo* organ culture of mouse tibias and immunofluorescent staining (Figure 7). The expression of C/EBPβ was increased by the infection of adenovirus vector expressing C/EBPβ-LAP, indicating that transfection of C/EBPβ was effectively performed. As we previously reported, hypertrophic transition of cultured tibias was observed in morphology as well as protein expression [19]. The expression of Ihh and RUNX2 was increased in the tibias which were transfected with C/EBPβ, suggesting that C/EBPβ regulates the expression of Ihh.

## Discussion

Chondrocyte differentiation is tightly regulated by various factors. Several studies have shown that C/EBPβ is one of the transcription factors involved in regulating hypertrophic differentiation of chondrocytes during skeletal development [16], [19]. Meanwhile, the Ihh/PTHrP negative feedback loop is reported to be an important mechanism to control the pace of differentiation from proliferative to hypertrophic chondrocytes [4]. The present study is the first to show that C/EBPβ stimulates the expression of *Ihh* during chondrocyte differentiation by directly binding to its promoter region. Furthermore, the binding element of C/EBPβ is also important for RUNX2 to activate *Ihh*.

Overexpression of C/EBPβ stimulated the expression of *Ihh* as well as *Pthrp* (Figure 2). This stimulation of *Pthrp* expression might be caused by increased *Ihh* expression. In contrast, knockdown of C/EBPβ had the opposite effect on *Ihh* expression (Figure 3). Decrease of *Ihh* expression was only observed on the 14th day because ATDC5 cells intrinsically exhibit endogenous *Ihh* expression at these late stages of culture. The expression of *Pthrp* was stimulated by the C/EBPβ knockdown at the early stages of culture when the expression of *Ihh* did not change (Figure 3). Previously, we reported that *Cebpb* knockdown by shRNA in ATDC5 cells increased both mRNA and nuclear protein of SOX9 at day 4 [19]. It was also reported that PTHrP is a direct transcriptional target of SOX9 [28]. Therefore, the increased expression of *Pthrp* in ATDC5 cells transfected with shRNA for *Cebpb* may be caused by increased SOX9. These gain and loss of function experiments suggested that C/EBPβ is involved in the regulation of *Ihh*. In this study, therefore, we focused on the interaction between C/EBPβ and Ihh in chondrocytes.

*Ihh* has been shown to be regulated by several factors in chondrocytes. Activating transcription factor 4 (ATF4), a leucine zipper-containing protein of the cAMP response element-binding protein (CREB) family, directly up-regulates transcriptional activity of *Ihh* in chondrocytes [29]. RUNX2, with the assistance of RUNX3, regulates limb growth by organizing chondrocyte maturation and proliferation through the induction of *Ihh* expression [23]. It was also reported that BMP and Ihh/PTHrP signaling interact to regulate hypertrophic differentiation of chondrocytes [6]. Meanwhile, previous studies have demonstrated that C/EBPβ also interacts with other transcription factors to regulate expression of target genes. During the process of osteoblast maturation, C/EBPβ promotes the expression of osteocalcin cooperatively with ATF4 or RUNX2 [20], [21]. In the regulation of the *MMP13* gene, C/EBPβ is an important stimulator that cooperates with AP-1, which is a leucine zipper transcription factor [14]. Moreover, C/EBPβ stimulates the expression of *MMP13* by interacting with RUNX2 during chondrocyte differentiation and OA development [17]. In fact, C/EBPβ increased the expression of RUNX2 in differentiating ATDC5 cells [19]. In the present study, therefore, we focused on the cooperative binding of C/EBPβ and RUNX2 to the C/EBPβ binding element in the *Ihh* promoter. This study revealed that a point mutation introduced into the C/EBPβ binding element significantly weakened the stimulatory effect of RUNX2 on the promoter activity (Figure 6B). Considering with the results of EMSA, ChIP and IP (Figure 6C, D, E), C/EBPβ binding element, in addition to RUNX2 binding elements, is crucial not only for binding of C/EBPβ itself, but also for RUNX2 binding. In a previous study, however, deletion assay of mouse *Ihh* promoter and EMSA demonstrated the direct regulation of *Ihh* by RUNX2 through some other binding elements [23]. Our preliminary data also showed that RUNX2 stimulated luciferase activity of the *Ihh* reporter construct, but up-regulation of *Ihh* luciferase activity by RUNX2 was gradually weakened along with deletion of promoter elements (data not shown). Therefore, RUNX2 could regulate the expression of *Ihh* at multiple binding elements *in vivo*.

Endochondral ossification is also observed during osteoarthritic cartilage [2]. C/EBPβ as well as Ihh and its downstream signaling targets are known to be up-regulated in degraded cartilage [14], [15], [30], [31]. Pharmacological inhibition of hedgehog signal reduced the severity of OA by repressing ADAMTS5 through RUNX2 modulation [30]. Moreover, recombinant PTH(1-34) prevented progression of OA in rats *in vivo* presumably by PTH repressing Ihh expression and inhibiting hypertrophic differentiation of chondrocytes [32]. This study revealed that C/EBPβ regulates *Ihh* expression upstream of hedgehog signaling, suggesting that C/EBPβ could be a therapeutic target for OA.

C/EBPβ has been reported to regulate various genes during chondrocyte differentiation and OA development. We recently reported that C/EBPβ represses the expression of *Col2a1* and *Sox9* during chondrocyte differentiation [19]. C/EBPβ also promotes hypertrophic differentiation of chondrocytes by regulating *Col10a1* [18] or *p57*, which is known to be a cell cycle factor [16]. We have also shown that C/EBPβ, induced by the pro-inflammatory cytokines such as IL-1β and tumor necrosis factor α (TNFα), stimulated the expression of MMP3 [15] and MMP13 [14] and repressed the expression of *Cd-rap* [13] in OA cartilage. Thus, C/EBPβ has multiple functions in chondrocytes of arthritic cartilage that exhibit matrix degradation and hypertrophic transition of chondrocytes.

## Conclusions

Our present study demonstrates that C/EBPβ directly regulates the expression of *Ihh* during differentiation from proliferative to hypertrophic chondrocytes. In addition, RUNX2 stimulates the transcriptional activity of *Ihh* through the C/EBPβ binding element. Therefore, C/EBPβ plays multiple roles in matrix degradation and chondrocyte differentiation during bone development as well as in arthritic cartilage.
